# Supplementary material for: Health shocks, medical insurance and household vulnerability: Evidence from South Africa
Source: PLoS One. 2020 Feb 7;15(2):e0228034. doi: 10.1371/journal.pone.0228034 (PMC7006899; doi:10.1371/journal.pone.0228034)
Supplement: S1 Table — (DOCX) [file pone.0228034.s001.docx]

| S1 Table: Description of Variables | | |
| --- | --- | --- |
| Variable | Description | Type |
| Food expenditure shock | Identifier of whether the household experienced a significant decrease in per capita real total food expenditure or not. | Dummy |
|  | Y= 1 if the decrease in per capita food expenditure was more than the standard deviation of per capita real food expenditure |  |
|  | Y=0 if otherwise |  |
| Health shock | Identifier of whether the head of the household experienced a significant decrease in BMI change or not | Dummy |
|  | X=1 if the decrease in BMI change was more than one standard deviation of the BMI-change distribution |  |
|  | X=0 if otherwise |  |
| HH Income | Real monthly household total income per capita in thousands. Deflated with StatsSA consumer price index data using December 2012 CPI as a base. In rands. | Continuous |
| HH Medical Expenditure | Real monthly household total medical expenditure per capita in thousands. Deflated with StatsSA consumer price index data using December 2012 CPI as a base. In rands. | Continuous |
| HH food expenditure | Real monthly household total food expenditure per capita in thousands. Deflated with StatsSA consumer price index data using December 2012 CPI as a base. In rands. | Continuous |
| Logged Food exp | Logged real monthly household total food expenditure | Continuous |
| Education attainment | Years of education completed by the household head | Continuous |
| Education: Primary | X=1 if years of education completed is between 1 and 5  X=0 otherwise | Dummy |
| Education: Secondary | X=1 if years of education completed is between 6 and 11  X=0 otherwise | Dummy |
| Education: Certification | X=1 if years of education completed is 12  X=0 otherwise | Dummy |
| Education: University | X=1 if years of education completed is greater than 12  X=0 otherwise | Dummy |
| Urban | Identifier of whether the household' dwelling is located in urban area or in traditional dwelling and farms | Dummy |
|  | X=1 if household is in urban location |  |
|  | X=0 if otherwise |  |
| Employed | Identifier of whether self-employed or employee | Dummy |
|  | X=1 if self-employed or employee |  |
|  | X=0 if otherwise |  |
