# Supplementary material for: Health shocks, medical insurance and household vulnerability: Evidence from South Africa
Source: PLoS One. 2020 Feb 7;15(2):e0228034. doi: 10.1371/journal.pone.0228034 (PMC7006899; doi:10.1371/journal.pone.0228034)
Supplement: S2 Table — (DOCX) [file pone.0228034.s002.docx]

| S2 Table: Parallel Trend Assumption tests | | |
| --- | --- | --- |
|  | Whole sample | Whole sample categories |
| Post | 0.4647** | 0.4475** |
|  | (0.1824) | (0.1797) |
| Period 1*Treat | -1.1620 | -1.2879 |
|  | (1.0554) | (1.0731) |
| Period 3*Treat | 1.9818** | 1.8711** |
|  | (0.9432) | (0.9469) |
| Treat | -1.0316 | -1.0633 |
|  | (0.6696) | (0.6763) |
| Medical Aid coverage | -0.2727 | -0.4333 |
|  | (0.3212) | (0.3152) |
| Female | -0.2823 | -0.3524** |
|  | (0.1868) | (0.1785) |
| Married | 0.1584 | 0.1315 |
|  | (0.2336) | (0.2350) |
| Age | -0.0059 | - |
|  | (0.0104) |  |
| 30-49 |  | 0.0529 |
|  |  | (0.3383) |
| 50-64 |  | -0.1956 |
|  |  | (0.3848) |
| 65-91 |  | 0.4343 |
|  |  | (0.5811) |
| Education Attainment | -0.0138 | - |
|  | (0.0213) |  |
| Primary |  | 0.4557* |
|  |  | (0.2557) |
| Secondary |  | -0.1051 |
|  |  | (0.3054) |
| Certificate |  | -0.2333 |
|  |  | (0.4082) |
| Undergraduate |  | 0.4736 |
|  |  | (0.4234) |
| Postgraduate |  | -1.2028 |
|  |  | (0.8319) |
| Household size | 0.1082*** | - |
|  | (0.0259) |  |
| 4-6 |  | 0.6898*** |
|  |  | (0.1909) |
| 7-9 |  | 0.6027** |
|  |  | (0.2397) |
| 10-24 |  | 0.8829*** |
|  |  | (0.3131) |
| Pension recipient | -0.2513 | -0.6967* |
|  | (0.2818) | (0.3759) |
| Urban | 0.1656 | 0.1353 |
|  | (0.1685) | (0.1688) |
| Employed | -0.3202 | -0.2981 |
|  | (0.1972) | (0.2010) |
| Constant | -2.0806*** | -2.3946*** |
|  | (0.5351) | (0.4143) |
| Observations | 4770 | 4771 |
| Note: *** Significant at 1 percent level, ** significant at the 5 percent level, * Significant at the 10* level | | |
