# Supplementary material for: Health shocks, medical insurance and household vulnerability: Evidence from South Africa
Source: PLoS One. 2020 Feb 7;15(2):e0228034. doi: 10.1371/journal.pone.0228034 (PMC7006899; doi:10.1371/journal.pone.0228034)
Supplement: S3 Table — (DOCX) [file pone.0228034.s003.docx]

| S3 Table: PS-Matching T-Test Results - Logit | | |  |  |  |  |  |
| --- | --- | --- | --- | --- | --- | --- | --- |
|  |  | Mean | |  | \|%\| Bias | t-test | |
| Variable | sample | Treated | Control | % Bias | Reduction | t | p>\|t\| |
| HH logged Income | Unmatched | 6,8815 | 6,9697 | -8,5 |  | -0,36 | 0,965 |
|  | Matched | 6,8612 | 6,8556 | 0,5 | 94.1 | 0,04 | 0,965 |
| Logged Food Exp | Unmatched | 5,4468 | 5,4687 | -2,6 |  | -0,11 | 1 |
|  | Matched | 5,4102 | 5,4102 | 0 | 100 | 0 | 1 |
| Medical Aid Coverage | Unmatched | 0,05 | 0,05 | 0 |  | 0 | 0,584 |
|  | Matched | 0,06612 | 0,04959 | 5,9 | - | 0,55 | 0,584 |
| African | Unmatched | 0,9 | 0,9 | 0 |  | 0 | 0,352 |
|  | Matched | 0,90083 | 0,93388 | -10,2 | - | -0,93 | 0,352 |
| Age 2 | Unmatched | 3246,8 | 3096,3 | 9,6 |  | 0,43 | 0,482 |
|  | Matched | 3222,6 | 3069,1 | 9,7 | 1.0 | 0,7 | 0,482 |
| Illness | Unmatched | 0,25 | 0,2 | 11,5 |  | 0,53 | 0,671 |
|  | Matched | 0,29752 | 0,27273 | 5,6 | 51.3 | 0,43 | 0,671 |
| HH Food Expenditure | Unmatched | 362,28 | 388,52 | -5,6 |  | -0,19 | 0,833 |
|  | Matched | 323,61 | 313,46 | 2,4 | 57.1 | 0,21 | 0,833 |
| Female | Unmatched | 0,65 | 0,575 | 15,7 |  | 0,68 | 0,429 |
|  | Matched | 0,64463 | 0,59504 | 10,4 | 33.8 | 0,79 | 0,429 |
| Mean Bias | Unmatched | 6.7 |  |  |  |  |  |
|  | Matched | 5.6 |  |  |  |  |  |
| *B>25% | Unmatched | 24.2 |  |  |  |  |  |
|  | Matched | 23.3 |  |  |  |  |  |
| * R outside [0.5; 2] | Unmatched | 0.93 |  |  |  |  |  |
|  | Matched | 0.58 |  |  |  |  |  |
|  |  |  |  |  |  |  |  |
