# Supplementary material for: Health shocks, medical insurance and household vulnerability: Evidence from South Africa
Source: PLoS One. 2020 Feb 7;15(2):e0228034. doi: 10.1371/journal.pone.0228034 (PMC7006899; doi:10.1371/journal.pone.0228034)
Supplement: Attachment — Submitted filename: Foster_review_2019_0925.docx [file pone.0228034.s004.docx]

**Reviewer: Nicola Foster**

**Manuscript number: PONE-D-19-21685**

**Title: Health shocks, medical insurance and household vulnerability: evidence from South Africa**

Thank you for the opportunity to review the manuscript *Health shocks, medical insurance and household vulnerability: evidence from South Africa*.

It is really great to see the innovative ideas presented in this paper, and the questions identified are highly relevant for South Africa (and globally) for debates related to conceptualising Universal Health Coverage. The comments presented are aimed at strengthening the analysis presented here, and more broadly the conclusions drawn from the work. Comments are structured following the sections of the paper.

Overarching

- Currently, the manuscript reads as if written for an economics journal, and while this is not a problem, I would recommend that in order to make the paper more accessible to a wider audience, there is a need to use a style that is similar in structure to biomedical papers typically published in PLoS ONE. For each of the sections of the manuscript, I offer suggestions that may assist in improving this.
- In terms of the structure of the manuscript, perhaps you could consider amending headings to for example “measuring health shocks and household vulnerability” to “analytical framework”, “econometric strategy” perhaps rather as “methods”. Section 5. Data could possibly be subdivided to have a section “main results” and “robustness checks”. “6. Conclusion” could be renamed as “Discussion and Conclusion” as there is a decent proportion of section 6 that is in fact a detailed discussion of the results of the study.

Abstract

- Suggest rewriting the abstract to follow a format with introduction, methods, findings and conclusions. Currently, the paragraph opens with the objective of the study which feels out of place. It would improve the reading if you started with the global background to the study, for example the current debates around Universal Health Coverage (UHC).
- From the rest of the paper, I am not convinced that households are in fact showing a preference for private healthcare – is it possible that this is rather what they have access to? While all taxpayers (including through VAT) contribute to the financing of the public health sector in South Africa, those who are in formal employment are often enrolled into private health insurance by their employers.
- Please substantiate statement “the results confirm that access to quality healthcare is a privilege in South Africa”. It would be helpful if you could expand on how the quality of healthcare was conceptualised in this study and how implemented in this analysis?

Introduction

- Suggest adding a section that explicitly describes the characteristics of the South African health system. With an emphasis on the arrangement of and funding of health services between the public and private health system.
- If preferences for services (and how this intersects with affordability) is a thread that you would like to further explore in your work, then I suggest that this needs to be unpacked in more detail.
- It would be helpful to explicitly discuss out-of-pocket payments as one of the funding mechanisms for healthcare in South Africa – and how the burden of out of pocket payments affects people from different socio-economic status groups who are accessing health services from the public – and private health sectors. Perhaps you can also further expand on the funding for private health care services, which will include private health insurance, but also out of pocket payments? And perhaps a bit more on the range of benefit packages offered by various private health insurance schemes. For example, would households experience the same burden of out-of-pocket payment irrespective of the insurance scheme that they belong to? This would set up the introduction to your study better.

On page 3:

- In the figures related to medical aid coverage quoted, please specify enrolment in health insurance by type of insurance.
- Please provide a reference for the poverty rates quoted, and a definition for how poverty was defined here.
- Would be helpful to update the current developments related to the National Health Insurance in South Africa, especially the White Paper. Could you please reference the statement that the delays are related to sufficient public funding?
- Could you expand on your literature review related to vulnerability and food expenditure? In particular, could you unpack the direction of the relationships that have been explored in the literature you reviewed? One review to perhaps consider/ that may assist in identifying additional literature sources is Russell. 2004. [The economic burden of illness for households in developing countries: a review of studies focusing on malaria, tuberculosis and HIV](http://www.ajtmh.org/docserver/fulltext/14761645/71/2_suppl/0700147.pdf?expires=1569423051&id=id&accname=guest&checksum=910D88FA7355BC2611A34B76FED14364). In his review, he found that some of the studies relying on primary data collection, showed that household expenditure on food may increase when ill – but I think the question as to whether this may mean that there are intra-household shifts in food expenditure is less clear.
- The last paragraph before section 2, starting with “Given the binary nature of the dependent variable…”, would probably best fit in your main results section.
- On the definition of health shocks, I agree with the use of reductions in BMI as a measure, following the work of Wagstaff in Vietnam, however it would be helpful to expand a bit on your explanation of this measure. Could you say something about how relevant the work from Vietnam is likely to be to South Africa? Is this measure transferable and relevant, especially given the mixed evidence related to association and trend in South Africa between poverty and type II diabetes which is associated with higher BMIs? For example, Mutyambizi et al. 2019. Lifestyle and socio-economic inequalities in diabetes prevalence in South Africa: a decomposition analysis – find some evidence of an association between diabetes and higher SES in South Africa. However, Ataguba in an analysis of panel data, found a reduction in this trend over time. Is the focus on non-obese household heads, an attempt to control for the association with Diabetes, if so, please specify?

On page 6:

- Under econometric strategy, could you please explain here in which ways your study used a quasi-experimental data design?

On page 7:

- Given, the audience, it would be helpful to rewrite the sentence beginning with “Furthermore, given the panel data structure…” in simpler language. Especially, given that reverse causality could be used to wither mean that the direction of the cause and effect relationship is here the opposite of what one would normally consider, OR that there is a two-way causal relationship. I think that you mean the latter, but it would be helpful to be clear.
- With your difference-in-difference explanation, could you first succinctly explain what the first difference being assessed is; and then the second. An additional explanation would again assist readers without an economics background to understand your study better. Ideally this explanation should come before the equation (number 3) on page 7. Could you add a sentence to explain why you are using a non-linear DiD specifically with your question in mind?

On Page 10:

- Here, it would be worth reminding the reader that a health shock is defined as a significant reduction in BMI.

On Page 11:

- Table 2 would read better if you presented the comparison between Treated and Control in columns.
- Please be consistent in reporting the number of decimal points.
- Would suggest defining food shocks and how measured in the text here.
- In the table/ below the table, it would be helpful to repeat the definition of who the treated and control groups are again, i.e. those with a health shock vs those not.

Page 12:

- Remind the reader what a quintile one household is in the text. While it seems redundant, it really assists the readability.
- The reporting of changes between the groups could be improved by being consistent in reporting the decimal positions.
- Could you be specific about the threshold or the SA government pension grant (plus add a reference). How does that relate to the mean household income for each of your quintiles?

Page 13:

- In Table 3, again, it might be helpful to put the comparators (i.e. quintile 1 and quintile 2) on the horizontal axis; with the sample characteristics reported on the vertical axis.
- In your labelling, for each of the variables, it would be helpful if you could also specify the time period. So, for example, total medical expenditure per month? Per annum? In addition, please make sure that you report to the same number of decimal points.
- Page 13 (Table 3), could you please also discuss the finding that richer households has much greater total medical expenditure?
- Is “Head education attainment” a categorical variable? Or number of years of schooling? Please, clarify in the text.
- It would be helpful to also explain how medical aid coverage is defined i.e. does this include all patients who are accessing any form of health insurance whether it is full coverage or hospital only?

Tables 5, 6 and 7:

- For the reader, it would really improve the ability to see what is going on in the tables if you would add what the comparators are that are being reported.

Page 14:

- It would be helpful to substantiate from your results, why you conclude that “higher income households respond to health shocks by reallocating household spending towards the sick household member…”
- You may need to expand on how your results show that “higher income households have a preference to access better quality private sector healthcare”.
- Could your finding that poorer households do not have a reduction in food expenditure in the face of health shocks not also be explained by the protective effect of fee-free public health services in South Africa?
- Agree that this affect might be mediated by informal community support systems, and donations, but could it also be affected by labour behaviour. In the absence of out of pocket costs for healthcare, we would suspect that the largest cost contributor to health shocks would be loss of income. However, perhaps in South Africa (and especially in those informally employed) it might be that even significant reductions in BMI would not lead to a loss of income.

Page 19:

- The discussion/ conclusion section is well explained and points well argued.
